# The Inhibitory Effects of Phenolic and Terpenoid Compounds from *Baccharis trimera* in Siha Cells: Differences in Their Activity and Mechanism of Action

**DOI:** 10.3390/molecules180911022

**Published:** 2013-09-09

**Authors:** Cristiane B. de Oliveira, Lucimara N. Comunello, Érica S. Maciel, Scheron R. Giubel, Alessandra N. Bruno, Eduardo C. F. Chiela, Guido Lenz, Simone C. B. Gnoatto, Andréia Buffon, Grace Gosmann

**Affiliations:** 1 Laboratório de Fitoquímica e Síntese Orgânica (LAFIS), Faculdade de Farmácia, Universidade Federal do Rio Grande do Sul (UFRGS), Porto Alegre 90610-000, RS, Brazil; E-Mails: cristiane_gabi@yahoo.com.br (C.B.O.); maracomunello@hotmail.com (L.N.C.); ericascor@bol.com.br (É.S.M.); simone.gnoatto@ufrgs.br (S.C.B.G.); 2 Laboratório de Análises Bioquímicas e Citológicas (LABC), Faculdade de Farmácia, Universidade Federal do Rio Grande do Sul (UFRGS), Porto Alegre 90610-000, RS, Brazil; E-Mail: andreia.buffon@ufrgs.br; 3 Instituto Federal de Educação, Ciência e Tecnologia, Porto Alegre 90030-041, RS, Brazil; E-Mails: scheron_rathke@hotmail.com (S.R.G.); alessandra.bruno@poa.ifrs.edu.br (A.N.B.); 4 Laboratório de Sinalização e Plasticidade Celular, Departamento de Biofísica, Universidade Federal do Rio Grande do Sul (UFRGS), Porto Alegre 91501-970, RS, Brazil; E-Mails: eduardochiela@gmail.com (E.C.F.C.); lenz@ufrgs.br (G.L.)

**Keywords:** phenolic compounds, terpenoids, cervical cancer, necrosis, apoptosis, *Baccharis trimera*

## Abstract

*Baccharis trimera* is used in folk medicine as a tea for digestive and liver diseases. It possesses anti-inflammatory and antioxidant properties that are related to the presence of phenolic compounds. The aim of this work was to investigate the anti-proliferative properties of phenolic (PHE) and terpenoid (SAP) compounds from *B. trimera* on human cervical cancer. The treatment of SiHa cells with PHE for 24 h suppressed colony formation in a dose-dependent manner, inhibited proliferation and inhibited cell motility. Although SAP inhibited the proliferation of SiHa cells in a dose-dependent manner, it increased colony formation and did not inhibit cell motility. PHE and SAP also promoted a significant increase in lactate dehydrogenase levels in the culture medium in a dose-dependent manner, indicating a loss of cell membrane integrity. Moreover, PHE promoted necrotic cell death, whereas SAP induced apoptosis. These compounds are new anticancer prototypes due their significant anticancer activity demonstrated herein.

## 1. Introduction

Cervical cancer is an important public health problem worldwide [1] due to the variable efficacy of available treatments, possibility of recurrence, high cost of care and a number of side effects with great influence on a patient’s quality of life. Moreover, some types of cervical cancer do not respond well to treatment, and the recurrence of cervical cancer constitutes a major clinical problem [2]. Due to the importance of plants as a source of new drugs, the search for active plant-derived compounds as prototypes for new anticancer therapies is necessary.

The tea prepared from *Baccharis trimera* (which is popularly known as “carqueja”) is used in folk medicine to treat digestive and liver diseases [3]. The chemical composition of *B. trimera* reportedly consists of flavones, flavonols, saponins and diterpenes [4,5]. The phenolic compounds previously identified in *B. trimera* include apigenin, 7,4′-di-*O*-methyl-apigenin, cirsimaritin, eupatorin, genkwanin, hispidulin, isoquercetin, luteolin, nepetin, quercetin, 3-*O*-methylquercetin, 5,6-dihydroxy-7,3′,4′-trimethoxyflavone and rutin. In relation to terpenoids, *B. trimera* present mainly saponins, among which echinocystic acid is the major aglycone [6,7,8]. Many biological activities, such as anti-inflammatory, antioxidant, analgesic, anti-hepatotoxic and muscle relaxant effects, have been ascribed to *B. trimera* [6,7,9,10]. Our previous studies have shown that the phenolic compounds (PHE, 15 mg/kg) of *B. trimera* exhibit anti-inflammatory activity using a pleurisy model in rats treated with carrageenan. PHE also has antioxidant effects similar to those of vitamin C and the flavonoids quercetin and luteolin, as measured by the DPPH assay [11].

Compounds with anti-inflammatory and antioxidant activities play an important role in antitumor activity. Oxidative stress may cause damage to DNA, thus inducing mutations that may contribute to progressive tumor growth [12]. Several reports have demonstrated the anti-proliferative effects of herbal polyphenols, such as quercetin, in various human cancer cell lines [13,14,15]. Considering the anticancer activity of phenolic compounds, we chose to examine the effects of phenolic (PHE) and terpenoid (SAP) compounds derived from *B. trimera* on SiHa cells, a cervical cancer cell line.

## 2. Results and Discussion

### 2.1. Reduction of SiHa Cell Viability

**Figure 1:**
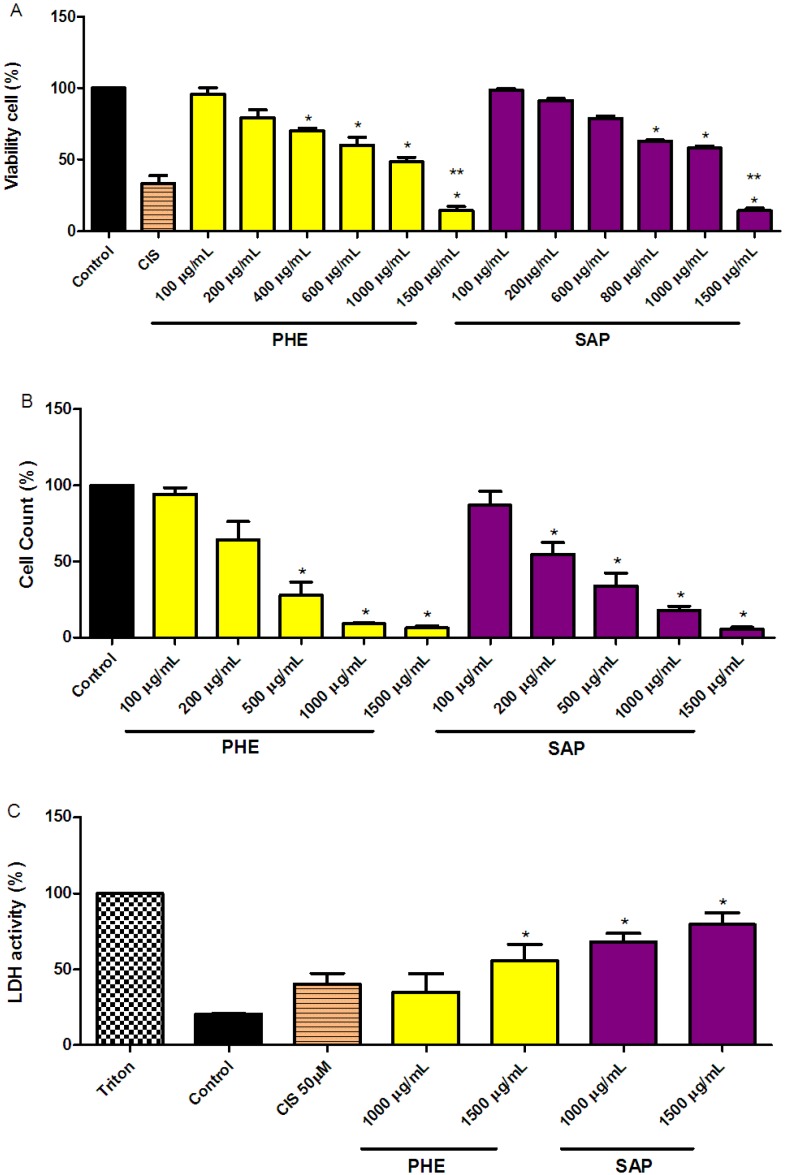
SiHa cell viability effects. (**A**) The cells were incubated with PHE or SAP for 24 h; (**B**) Cell counts after treatment with PHE or SAP for 24 h; (**C**) Loss of membrane integrity measured by LDH release after treatment with PHE or SAP for 24 h. All values are the means ± SE of at least triplicate cultures in four independent experiments (* p < 0.05 as compared to the control and ** p < 0.05 compared to cisplatin (CIS) 50 µM).

The MTT assay results showed a dose-dependent anti-proliferative effect after 24 h of treatment for both PHE and SAP. At 1,500 mg/mL, the reduction in cell viability was 86% for both PHE and SAP. A 50 mM cisplatin treatment (one of the drugs used to treat cervical cancer) reduced cell viability to 62% ± 11.16% (Figure 1A). The decrease in cell viability was confirmed using a cell-counting assay (Figure 1B). The inhibitory concentration (IC_50_) was found to be 482 µg/mL and 456 µg/mL for PHE and SAP, respectively. 

Both PHE and SAP were previously identified as potentially active compounds in several models of antioxidant *in vitro* assays and anti-inflammatory *in vivo* models [9,10,11]. Herein, PHE and SAP inhibited over 80% of cellular growth in a dose-dependent manner compared to the control treatment. 

### 2.2. LDH Measurements in SiHa Cervical Cancer Cells

Compared to the control treatment (DMSO 0.3%), an increase in the activity of LDH was observed after 24 h of treatment with PHE or SAP. PHE and SAP (1,000 and 1,500 µg/mL, respectively) both promoted a significant leakage of LDH into the culture medium in a dose-dependent manner (Figure 1C), indicating a loss of cell membrane integrity.

### 2.3. Effects on Clonogenic Survival

**Figure 2:**
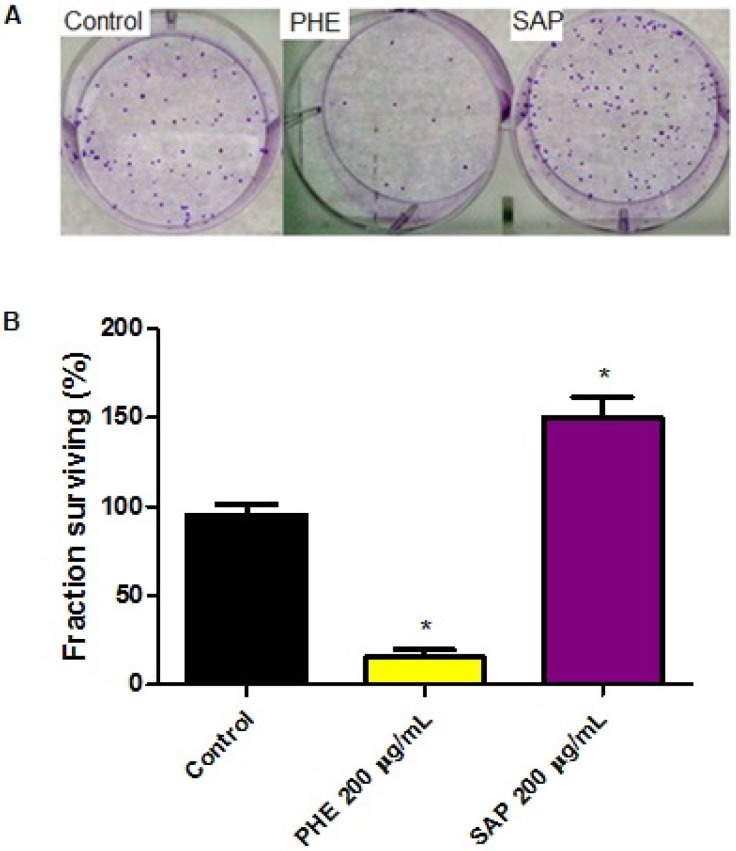
Effects on the clonogenic potential of tumor cells after exposure to 200 µg/mL of PHE or SAP. (**A**) Colonies formed after 24 h of treatment with PHE or SAP; 88 colonies were formed by untreated cells. After PHE treatment, 10 colonies formed; after SAP treatment, 135 colonies formed; (**B**) All values are the means ± SE of at least triplicate cultures in four independent experiments (*p* < 0.05 compared with the control).

SiHa colonies were evaluated after 10 days of treatment at 200 µg/mL (Figure 2). PHE decreased the clonogenic survival to 4% ± 0.57 with an SF = 0.20 compared to the control (DMSO 0.3%) treatment. However, SAP increased the number of colonies observed, with an SF = 1.48. These results indicate that phenolic and terpenoid compounds lead to cell death through different mechanisms of action.

### 2.4. Wound Healing Migration Assay

**Figure 3:**
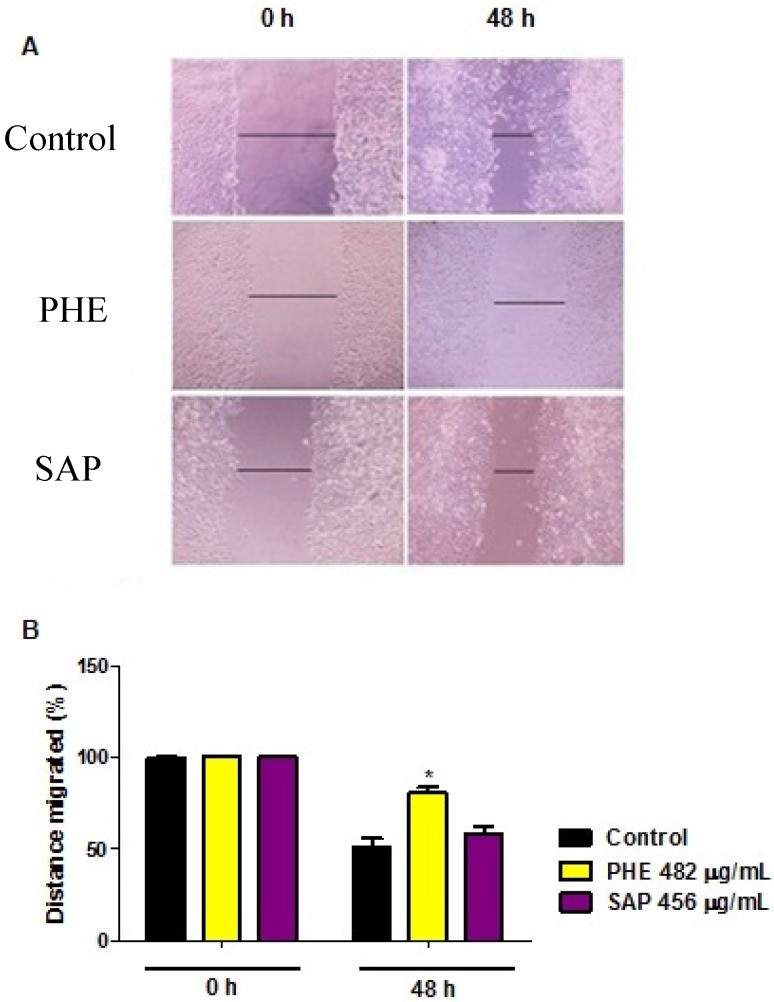
Wound-healing assay after treatment with the IC_50_ concentration of PHE (482 µg/mL) or SAP (456 µg/mL) for 24 h. (**A**) Confluent cultured cells were carefully wounded, incubated in RPMI serum-free medium and treated with or without compounds for 24 h, and the complete medium was then replaced. Cells were photographed using an inverted microscope at the indicated times; (**B**) Distance of migrated cells (% Control). All values are the means ± SE of at least triplicate cultures in four independent experiments (*p* < 0.05 compared with the control at 0 h and 48 h).

The ability of phenolic compounds and terpenoids to reduce cellular migration was investigated using a classic *in vitro* wound-healing assay. Cells were exposed to the IC_50_ concentration of each compound for 24 h. Figure 3 shows a representative experiment at times 0 and 48 h after wound initiation for both treated and untreated cells. In this experiment, the PHE treatment clearly reduced cell motility (approximately 20%). However, SAP did not affect cell migration compared to the control treatment.

### 2.5. Apoptosis Assay

Because PHE and SAP induced cell death in a dose-dependent manner (Figure 1), the necrotic and/or apoptotic cell population was determined by annexin-V/PI staining. Apoptosis and necrosis represent two fundamental types of cell death. Apoptosis plays an important role in the homeostasis of different tissues in response to numerous stimuli. This process is characterized by several morphological and biochemical changes in cells. It may take hours or days and is crucial for embryonic development, maturation of the immune defense against viral infections and tumor elimination. In contrast, necrosis occurs suddenly and is characterized by mitochondrial and cellular swelling following plasma membrane disruption [16,17,18,19].

**Figure 4:**
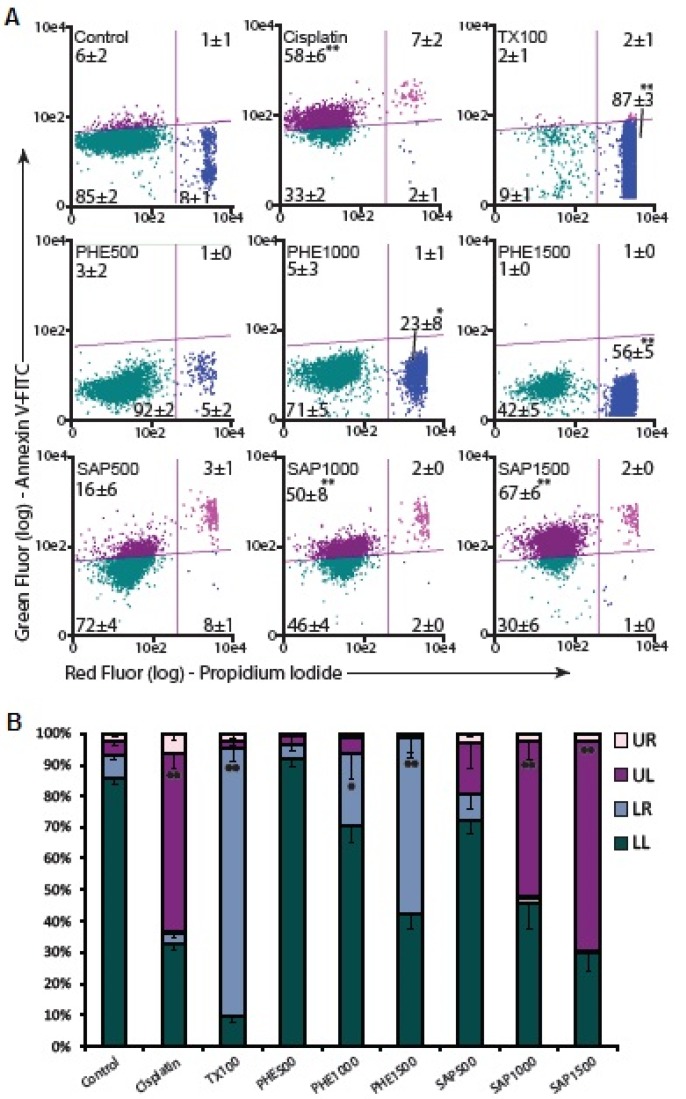
(**A**) Scatter plots of annexin-V-FITC/PI stained control cells and cells treated with PHE or SAP categorized by quadrant analysis. UL (upper left) early apoptotic cells, UR (upper right) late apoptotic cells, LL (lower left) normal cells and LR (lower right) necrotic cells. Triton X-100 (TX100) was used as necrosis positive control and Cisplatin was used as apoptosis positive control; (**B**) The data are representative of four independent experiments. All values are the means ± SE of at least triplicate cultures in four independent experiments (* *p* < 0.05 and ** *p* < 0.01 compared with the control).

SAP induced a dose-dependent increase in the apoptotic cell population after a 24-h treatment with 500, 1,000 or 1,500 µg/mL (Figure 4). At the highest concentration of 1,500 µg/mL, PHE promoted necrotic cell death in 56% ± 5% of the cells, whereas SAP induced cell apoptosis in 67% ± 6% of the cells. Flow cytometry analysis of cells stained with annexin-V-FITC and PI showed that after PHE treatment for 24 h, a significant increase in the number of necrotic cells was observed. In contrast, there was a significant increase in early apoptotic cells after SAP treatment (Figure 4).

Cervical cancer is the third most frequently occurring cancer, and the presence of human papilloma virus (HPV) infection is among one of the most common causes [1,16]. It is already known that terpenoids induce cellular death by apoptosis in various tumor cells [20,21].

Among the polyphenols present in PHE [6,7,8], several studies have shown that compounds such as flavonoids induce cell death via apoptosis [15,22,23,24], and other work [13] has shown that quercetin induces necrosis and apoptosis in SSC-9 oral cancer cells. These data indicate that quercetin may induce an initial shock to the cells that results in necrosis, followed by a reorganization of the remaining viable cells that subsequently undergo apoptosis after prolonged treatment. Our results indicate that PHE and SAP obtained from *B. trimera* tea elicited cytotoxic effects on SiHa cells through different mechanisms of action.

## 3. Experimental

### 3.1. Plant Material

*Baccharis trimera* (Less.) DC aerial parts were collected in Rio Grande do Sul, Brazil, and identified by Prof. Dr. Sérgio Bordignon from Centro Universitário La Salle, Brazil. A voucher specimen is deposited at the Herbarium (ICN 189790) of the Universidade Federal do Rio Grande do Sul. Plants were air-dried and powdered.

### 3.2. Chemical

All chemicals were of analytical grade and were purchased from Sigma (Milwaukee, WI, USA), Fluka Chemie (Buchs, Switzerland) or Merck (Darmstadt, Germany). 

### 3.3. Extraction

Purified PHE (polyphenols) and SAP (terpenoids) were prepared and characterized as described elsewhere [11]. Briefly, the plant material was exhaustively and successively extracted using a Soxhlet apparatus and the appropriate solvent, which provided the corresponding dichloromethane, ethyl acetate and butanol extracts. The ethyl acetate and butanol extracts were pooled together and submitted to molecular permeation chromatography on Sephadex LH-20 (GE Healthcare Bio-Sciences AB, Uppsala, Sweden) using 99% ethanol to obtain PHE (26%) and SAP (65%).

### 3.4. Cell Culture

Human cervical carcinoma SiHa cells were obtained from the American Tissue Culture Collection (ATCC, Rockville, MD, USA). Cells was cultured in Dulbecco’s modified Eagle medium (DMEM) supplemented with 10% fetal bovine serum (FBS) and 0.5 U/mL penicillin/streptomycin at 37 °C, in a 5% CO_2_ atmosphere at 100% humidity.

### 3.5. Treatments

PHE and SAP were dissolved in dimethyl sulfoxide (DMSO) and culture medium, at a concentration of 2 mg/mL. After reaching sub-confluence (70%–80% confluence), the cells were exposed to PHE or SAP (100 to 1,500 µg/mL) for 24 h in DMEM. Cells treated with DMSO (0.3% final concentration) were used as a negative control.

### 3.6. Cell Proliferation Assay

The inhibition of cell proliferation by PHE or SAP was assessed using the MTT (3-[4,5-dimethylthiazol-2-yl]-2,5-diphenyl tetrazolium bromide) assay [25]. Briefly, SiHa cells (3 × 10^3^ cells/well in 190 μL medium per well) were seeded in a 96-well plate. After 48 h, the cells were treated with PHE or SAP (100 to 1,500 μg/mL). Cisplatin (CIS) was used as a positive control. The optical density of each well was measured at 630 and 560 nm on an Envision (PerkinElmer, Waltham, MA, USA) microplate reader. Four independent experiments were performed in triplicate for each test. The results were expressed as the percentage of cell viability where cells with no treatment were considered 100% viable.

### 3.7. Cell Counting

SiHa cells (2.5 × 10^4^ cells/well in 600 μL medium per well) were seeded in a 24-well plate. After 48 h, cells were treated with PHE or SAP (100 to 1500 μg/mL). After a 24-h treatment, the medium was removed. SiHa cells were washed with phosphate-buffered saline (PBS), and 200 µL of 0.25% trypsin/EDTA solution was added to detach the cells from the plate. The counting was performed using trypan blue and a hemocytometer. The results were expressed as a percentage of the control to obtain the corresponding IC_50_.

### 3.8. LDH Measurement

The loss of membrane integrity was evaluated via the lactate dehydrogenase (LDH) assay. The supernatant of cells treated with PHE, SAP or control (DMSO) was used for the enzymatic assay and employed a LDH Kit available from Labtest Diagnostica (Minas Gerais, Brazil). The results were expressed as a percentage of 1% Triton X–100 induced LDH release [26].

### 3.9. Clonogenic Assay

To determine the growth suppression effects, SiHa cells were treated with DMSO (0.3%, control), PHE (200 µg/mL) or SAP (200 µg/mL) for 24 h. After treatment, cells were washed with PBS and trypsinized. One hundred cells/well were seeded in a 6-well plate, and the colonies formed after 10 days were fixed with methanol, stained with crystal violet and counted manually. The result was expressed as the surviving fraction (SF), which was obtained by dividing the number of colonies formed after the treatment by the number of cells seeded × PE (Plating efficiency), where PE = (N° of colonies formed/N° of cells seeded) × 100 [27].

### 3.10. Wound Healing Migration Assay

SiHa cells were plated in 24-well plates (2.5 × 10^4^ cells/well in 600 μL medium per well) and cultured in medium containing 10% FBS. The confluent monolayer of cells was carefully wounded using a yellow pipette tip and cellular debris was removed by washing with PBS. Then, the wounded monolayer was incubated with PHE, SAP or DMSO (control) for 24 h. Cell migration into the wound area was observed by an Olympus IX71 inverted microscope at time zero and 48 h after the scratch [28].

### 3.11. Apoptosis Assay

SiHa cells (2.5 × 10^4^ cells/well in 600 μL medium per well) were seeded in a 24-well plate. After 48 h, cells were exposed to PHE or SAP (500 to 1,500 μg/mL) for 24 h. After treatment, SiHa cells were double stained with FITC-conjugated annexin-V and PI using an Annexin-V Apoptosis Detection kit (Santa Cruz Biotechnology, INC, Dallas, TX, USA) according to the manufacturer’s instructions. Cells were analyzed in a GUAVA EasyCyte cytometer, and the data were analyzed using the GUAVA software ExpressPlus (Guava Technologies, Hayward, CA, USA). Triton X-100 (TX100) was used as a necrosis positive control and cisplatin was used as an apoptosis positive control.

### 3.12. Statistical Analysis

Data are expressed as the means ± standard error, and statistical significance was determined by a One-Way Analysis of Variance (ANOVA, *p* < 0.05), followed by Tukey’s test for multiple comparisons. 

## 4. Conclusions

To our knowledge, this is the first report demonstrating that compounds from *B. trimera* induce death in cervical cancer cells. PHE and SAP exhibit different activities and mechanisms of action by inducing death via necrosis or apoptosis, respectively. They represent an alternative avenue of research for the development of new anticancer prototype drugs.
